# Design of a novel wide-angle Rotman lens beamformer for 5G mmWave applications

**DOI:** 10.1038/s41598-024-51733-0

**Published:** 2024-01-13

**Authors:** Abolfazl Azari, Anja Skrivervik, Hadi Aliakbarian

**Affiliations:** 1 Microwave and Antenna Group (MAG), Ecole Polytechnique Fédérale de Lausanne, Lausanne, Switzerland; 2 Faculty of Electrical Engineering, K.N. Toosi University of Technology, Tehran, Iran

**Keywords:** Electrical and electronic engineering, Engineering

## Abstract

The attachment of 5G with millimeter wave (mmWave) frequencies offers massive capacity and low latency to reveal the full 5G experiences. High directive gain and beamforming are considered essential for mmWave 5G systems. The main requirements of the beamforming network for 5G mmWave applications are the scanning coverage of ± 60° and SLL > 10 dB in a wide operational bandwidth over the standard 5G frequency bands. In this paper, a novel PCB-based wide-angle Rotman lens beamformer is designed, simulated, and successfully measured to meet the mentioned requirements for 5G mmWave applications. A comprehensive improved design methodology is provided for all components of the Rotman lens to reach a wide scanning angle, enhanced sidelobe level, and low scan loss. The end-fire Vivaldi antenna is selected as an array element for easy integration to the beamforming network as well as its capability to use in dual-polarization configuration. The proposed Rotman lens is operational in the 24–30 GHz frequency band covering 5G *n257*, *n258*, and *n261* frequency bands. The results show a nearly constant 8 beams across the whole bandwidth steering from − 53° to 53° in 15° increments to provide ± 60° coverage with the SLL > 10 dB and scan loss < 1.9 dB. The retrieved novelties from this work contain an effective design methodology for an optimized Rotman lens with wide-scan angle and low phase and amplitude error, non-uniform distribution based array ports, and integration with end-fire antenna for possible dual polarization and 2-D beamforming capabilities. The comparison of the proposed beamformer with the most recent works shows several advantages in terms of integrated structure and performances including bandwidth, wide scanning angle, SLL, and scan loss. With such performances, this beamformer can be used for various mmWave and 5G applications such as advanced antenna systems, massive MIMO systems, and hybrid beamforming systems.

## Introduction

Millimeter wave (mmWave) has attracted huge interest in wireless technologies, in particular for the fifth generation of mobile communication (5G) applications in recent years. The demand for massive data rates and low latency in 5G systems can be addressed with the large available bandwidth in mmWave frequencies. The Advanced Antenna System (AAS) is a recommended necessity for 5G applications in mmWave frequencies. AAS employs a large antenna array with beamforming capability to improve the performance and spectral efficiency of 5G systems^1^. AAS requirements for 5G applications from the latest standards can be found in Ref.^1^ in terms of frequency bands, antenna element properties, array configurations, and beamforming characteristics. The 3rd Generation Partnership Project (3GPP) unites well-known standard organizations to develop protocols for mobile telecommunication systems^3^. Some standard mmWave frequency bands for 5G systems are^1–3^:

- *n257*: 26.5–29.5 GHz;
- *n258*: 24.25–27.5 GHz;
- *n261*: 27.5–28.35 GHz (subset of *n257*).

The single antenna element is recommended to be dual-polarized with the half power beam width of 65°^4^. The 5G antenna array known as AAS utilizes adaptive beam forming, multiple input multiple output (MIMO), and Spatial Division Multiple Access (SDMA)^1,2^. The main requirements of AAS for 5G mmWave applications are presented in Table 1.

**Table 1:** Main requirements of AAS^1^.

Operating frequency range	26.5–29.5 GHz 24.25–27.5 GHz 27.5–28.35 GHz
Array size	8 × 8
Polarization	Dual-polarized
Beamwidth of a single element (°)	65° for both H/V
Side lobe level (SLL)	> 10 dB
Scan angles in the horizontal plane	± 60°
Scan angles in the vertical plane	± 15°

Beamforming is a key technique in AAS that concentrates the power toward the desired direction and nulls the undesired directions. Beamforming can be implemented as analog, digital, and hybrid configurations. Analog beamforming is easy to implement but has a limited number and characteristics of fixed beams. Digital beamforming employs a separate RF chain for each array element, leading to a complicated structure, but provides very flexible and efficient beamforming. Hybrid beamforming utilizes both analog and digital beamforming as each RF chain is associated with multiple antenna elements^5,6^. The hybrid beamforming seems to be more efficient since the beamforming is performed in the analog domain using fewer RF chains.

It is clear that analog beamformers represent an important component of hybrid beamforming systems. Several different analog RF beamforming networks have been proposed mostly following the Butler matrix and Rotman lens topologies. The Butler matrix is a microwave network as the analog implementation of the fast Fourier transform including couplers and phase shifters. The phase shifts at the output ports can be determined by a combination of the phase shifts of all the signal paths. The Rotman lens is a scanning system that can be used in various systems as a fundamental multiple-beam antenna. In a Rotman lens, the required phase distribution on the antenna ports is achieved by true time-delay (TTD) through a shaping path and maintains a constant time delay over a broadband frequency range of operation^7^.

The Butler matrix is developed by integrating the couplers, phase shifters, and crossovers exhibiting significantly narrower bandwidths than most wideband antenna arrays. Rotman lenses are passive beamforming networks by implement true time-delay with relatively wideband performance^8^.

The main advantages of Rotman lenses compared to Butler and Blass Matrices are lower weight and hardware cost while having wider bandwidth and beam steering. Therefore, a Rotman lens is suitable for applications that require both a large scan of the radiation pattern and wide frequency range coverage^9^.

In Ref.^10^, a 5 × 8 Butler matrix operating at the frequency range of 27.8–30.8 GHz is presented where output signals with equal power divisions and five differential phases can be obtained. However, this beamforming network does not provide continuous beams.

A 16-element antenna array with a Butler matrix covering the band 26–31.4 GHz and ± 42° beam switching is presented in Ref.^11^. The maximum gain is 12 dBi with − 19 dB SLL, and 9 dBi with − 8 dB SLL for the beams with ± 13° and ± 42°, respectively.

In Ref.^12^ a wide angular Rotman lens operating in the 28 GHz band is proposed. The 6-port Rotman lens is connected to eight linear antenna arrays consisting of five series-fed rectangular patches. The angular scan is from − 60° to 60° with a variation of less than 8 dB. However, the bandwidth is low and the gain variation is high.

In Ref.^13^ a PCB-based Rotman lens consisting of an eight-element Yagi–Uda antenna array at 28 GHz is demonstrated. The proposed beamformer operates across 25.5–28.5 GHz with 7 switchable beams covering ± 30° with a realized gain of up to 9.4 dBi. However, the scan loss for the side angle (± 30°) is relatively high (4.5 dB).

With the extensive review of the literature on the mmWave Rotman lens topic, the following major problems in the existing designs can be recognized:

- Lack of Rotman lens design with wide-angle scanning around ± 60° over the wide frequency bandwidth.
- High scan loss for wide-angle beam compared to the central beam.
- Low SLL for wide-angle beam less than 10 dB.
- Lack of integration of the antenna with the beamforming network.

On the other hand, the Rotman lens beamforming network suitable for 5G AAS needs to be in line with the requirements indicated in Table 1. Therefore, the design of a Rotman lens with a minimum of 8 antenna elements with possible dual-polarization capability in a wide operational bandwidth over 5G frequency bands (24–30 GHz) for the scanning coverage of ± 60° and SLL > 10 dB is demanded by industry^1,2^.

In this research work, a novel wide-angle Rotman lens beamformer is developed to meet the AAS requirements for 5G applications. An exhaustive design methodology for the Rotman lens is extracted comprising different components of the Rotman lens including parallel-plate contour, beam ports, array ports, and dummy ports. For the antenna elements, an end-fire Vivaldi antenna in which the direction of radiation is along the line of the antenna is suggested for easy integration with the beamformer and facilitates dual polarization implementation as well as possible stacking to obtain 2-D beamforming. The beam ports and non-uniform array ports are designed in detail to accommodate good matching and enhanced SLL. A novel integrated matched load is also introduced for dummy ports. The designed beamformer is fabricated and tested to verify the results. A comparison between the proposed Rotman lens relative to the recent works is also provided.

The following merits can be summarized for the proposed Rotman lens beamformer taking into account addressing the mentioned problems with the existing designs.

- Optimized Rotman lens design methodology for wide scanning angle ± 53° covering ± 60° with 3 dB beamwidth.
- Low scan loss/dropping gain for the side beam (± 53°) less than 1.9 dB.
- Non-uniform antenna ports to satisfy the SLL > 10 dB for the side-beam (± 53°).
- End-fire antenna element integrated with the beamformer to eliminate the connector loss, possible dual-polarization, and stacking 2-D beamforming capabilities.
- PCB-based low-cost and high-performance beamformer covering multi 5G *n257*, *n258*, and *n261* frequency bands.

## Rotman lens beamformer design

**Figure 1:**
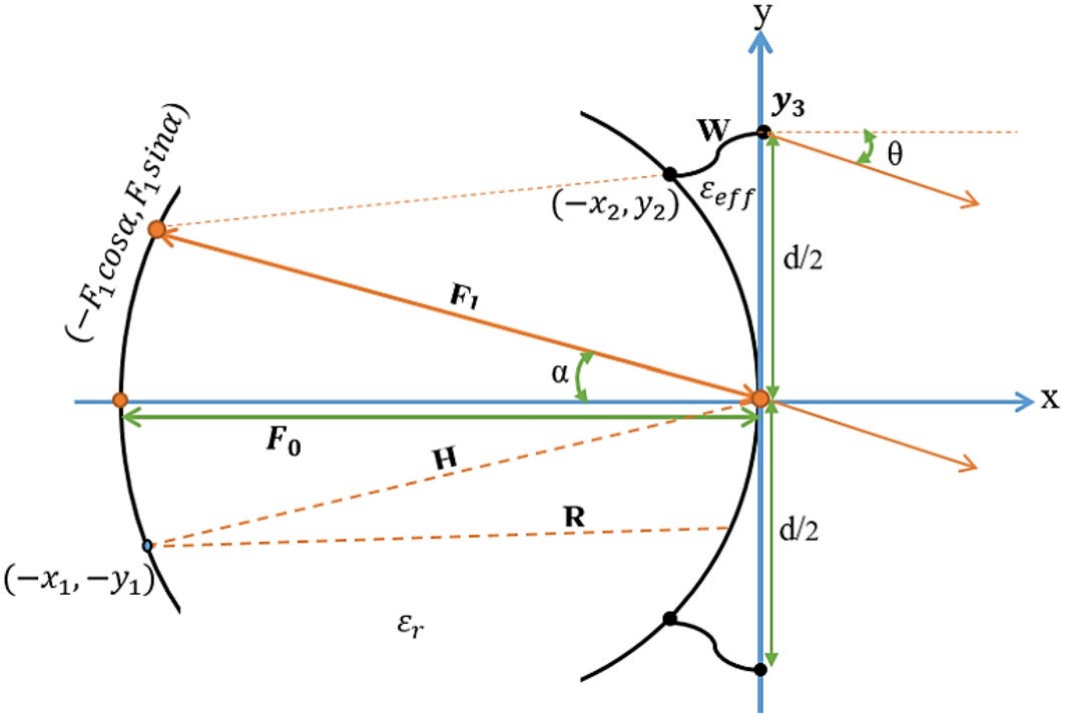
Schematic of a conventional Rotman-lens parameters.

The Rotman lens is a wide-angle lens that can be utilized as a wideband beamformer. The schematic of a conventional Rotman lens is shown in Fig. 1, which consists of a parallel-plate contour surrounded by *M*-beam ports and *N*-array ports. Each beam port steers the beam in a certain angular direction coming up with *M*-discrete beams. The array ports are connected via transmission lines to the radiating elements of a linear antenna array. The loaded dummy ports are connected to the parallel-plate region to provide an appropriate termination^14^.

The design of the Rotman lens starts with defining the general requirements of the beamformer such as the operating frequency range, the number of beam ports (*M*), the desired beam steering angle (± *θ*), the number of radiating elements (*N*) for specific gain performance, and the spacing between array elements (*d*)^15^.

The circular arc on the left side of the Rotman lens as the beam contour has the on-axis focal length ${F}_{0}$ located at 0° angle and the off-axis focal length ${F}_{1}$ located at angles α°. The general shape of the parallel-plate contour is determined based on the four basic Rotman lens parameters ${F}_{0}\cdot \alpha \cdot \beta \cdot \gamma$ as shown in Fig. 1.

Also, the length of transmission lines connecting each array-port to the lens (*W*) is essential for Rotman length design^14^.

Initial geometrical condition of the on-axis focal length ${F}_{0}$ for appropriate amplitude performance and also physical arrangments of the input and output ports, considering maximum scanning angle *θ* and array length (*N* − 1)*d*, can be defined as^16,17^:

1 $${F}_{0}\ge 2 \left(N-1\right)d {\text{ sin}}\theta .$$

The angle between the on-axis focal length ${F}_{0}$ and the off-axis focal length ${F}_{1}$ is the focal angle ($\alpha$) and the ratio between them is parameter $\beta :$

2 $$\beta ={F}_{1}/{F}_{0}.$$

The expansion factor $\gamma$ is the ratio between the focal angle and array beam angle as:

3 $$\gamma ={\text{sin}}\theta /{\text{sin}}\alpha .$$

The indirect factor of utility $\zeta$ controls the amplitude and phase error and corresponds to the distance of any point on the array from the axis (${y}_{3}$) to the on-axis focal length (${F}_{0}$) as expressed by:

4 $$\zeta ={y}_{3}\gamma /{F}_{0}.$$

The maximum distance of ${y}_{3}$ gives the maximum of the indirect factor of utility ${\zeta }_{max}$:

5 $${y}_{3({\text{max}})}=\left(N-1\right)d/2\to {\zeta }_{\text{max}}=(N-1)d\gamma /2{F}_{0}.$$

The upper limit of the indirect factor of utility appears when the transmission line *W* = 0 as:

6 $${\zeta }_{W=0}=\frac{2\sqrt{1-\beta C}}{S}\sqrt{1-\frac{1-\beta C}{{S}^{2}}},$$

$$C={\text{cos}}\alpha \cdot S={\text{sin}}\alpha .$$

**Figure 2:**
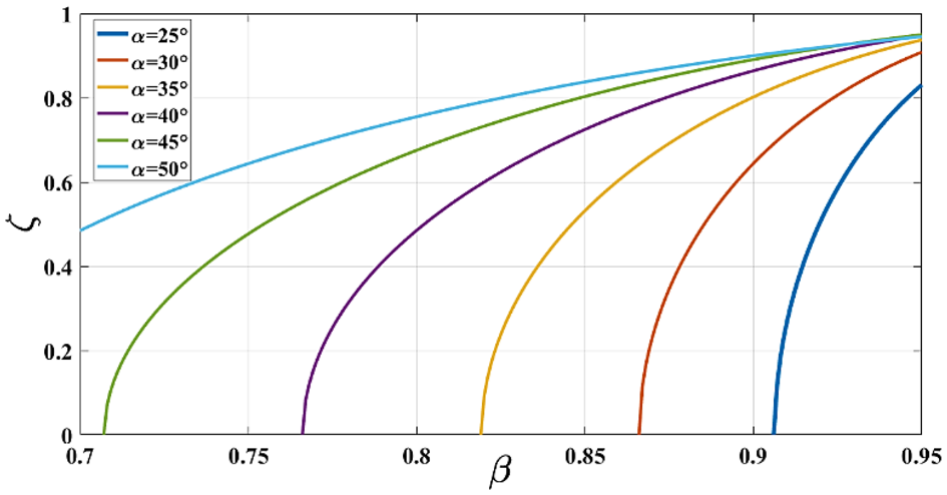
Limiting values of $\zeta$ versus $\beta$ for some $\alpha$ values.

The limiting value of the indirect factor of utility $\zeta$ versus $\beta$ is depicted for some focal angle values $\alpha$ in Fig. 2. Due to the fact that the useful range of $\zeta$ is between 0.5 and 0.8, Fig. 2 can be considered for choosing an appropriate range of $\beta$ for a given $\alpha$^15^.

In the case of fabricating the Rotman lens on a dielectric substrate with permittivity (${\varepsilon }_{r}$), all dimensions of the lens are divided by a factor of $\sqrt{{\varepsilon }_{r}}$.

The transmission line length that connects the element port to the array antenna can be calculated as^14^:

7 $$a{(W/{F}_{0})}^{2}+bW/{F}_{0}+c=0,$$

$$a=1-\frac{{\left(1-\beta \right)}^{2}}{{\left(1-\beta C\right)}^{2}}-\frac{{\zeta }^{2}}{{\beta }^{2}},$$

$$b=-2+\frac{{2\zeta }^{2}}{\beta }+\frac{2\left(1-\beta \right)}{1-\beta C}-\frac{{\zeta }^{2}{S}^{2}\left(1-\beta \right)}{{\left(1-\beta C\right)}^{2}},$$

$$c={-\zeta }^{2}+\frac{{\zeta }^{2}{S}^{2}}{1-\beta C}-\frac{{\zeta }^{4}{S}^{4}}{4{\left(1-\beta C\right)}^{2}},$$

$$C={\text{cos}}\alpha \cdot S={\text{sin}}\alpha .$$

The parameters $\alpha$ and $\beta$ have a comparable effect on Rotman lens geometry and significantly influence the gain performance and phase error. The parameters $\alpha$ and $\beta$ need to be selected in conjunction with other parameters to reach the optimized gain performance and phase error reduction.

The phase error is as a result of the path length difference between an arbitrary central ray through the center of array ports and any other arbitrary ray that can be evaluated as a function of scanning angle *θ* and indirect factor of utility $\zeta$. Thus, the normalized path length error $(\Delta l=\Delta L/{F}_{0})$ can be calculated as^14,17^:

8 $$\Delta l=\sqrt{{\varepsilon }_{r}}\left(r-h\right)+\sqrt{{\varepsilon }_{eff}}w+{y}_{3}{\text{sin}}\theta ,$$

where $h=H/{F}_{0}$ is the normalized distance from a point on the beam arc to the origin, $r=R/{F}_{0}$ is the normalized distance of any other point, $w=W/{F}_{0}$ is the normalized transmission line length, ${\varepsilon }_{r}$ is the dielectric constant, and ${\varepsilon }_{eff}$ is the effective dielectric constant of the transmission line as shown in Fig. 1. The total path difference stemming from all ports can be expressed as^14,17^:

9 $$\left|\Delta l\right|=\sum_{1}^{M}\sum_{1}^{N}\left|\sqrt{{\varepsilon }_{r}}\left(r-h\right)+\sqrt{{\varepsilon }_{eff}}w+{y}_{3}{\text{sin}}\theta \right|.$$

In order to estimate the amplitude performance, the approximation of the coupling between a beam port width ${WB}_{i}$ and array port width ${WA}_{j}$ can be presented as^17^:

10 $${S}_{{WB}_{i}{WA}_{j}}={J}_{0}\left(\frac{K{WB}_{i}}{2}{\text{sin}}{\phi B}_{i}\right){J}_{0}\left(\frac{K{WA}_{j}}{2}{\text{sin}}{\phi A}_{j}\right)\sqrt{\frac{{WB}_{i}{WA}_{j}}{\lambda d}}{e}^{-j\left(k{d}_{ij}+\pi /4\right)},$$

where $k=2\pi /\lambda$ is the phase constant, ${J}_{0}\left(x\right)=(sinx)/x$, ${d}_{ij}$ is the separation between ports *i* and *j*, and ${\phi B}_{i}$ and ${\phi A}_{j}$ are the angles between the boresight direction of ports and the line connecting the port phase centers.

The results of the Rotman lens design show that the minimization of the only path length error does not lead to the acceptable amplitude performance^17^. Therefore, the optimum performance of the Rotman lens can be achieved by considering both path length error and amplitude performance. Various optimization methods in particular numerical-based methods can be used for choosing $\alpha$ and $\beta$ optimally to reach reasonable phase error and amplitude performance simultaneously.

After designing the shape of the parallel-plate contour, the beam ports, array ports, and dummy ports should be designed. Firstly, the phase center of the corresponding ports should be determined and then the ports need to be matched with transmission lines.

The coordinate of the array port phase center $({x}_{2}\cdot {y}_{2})$ can be calculated as^18^:

11a $${X}_{2}=\frac{{x}_{2}}{{F}_{0}}=1-\frac{\frac{1}{2}{\zeta }^{2}{\text{sin}}^{2}\alpha +\left(1-\beta \right)W}{1-\beta {\text{cos}}\alpha },$$

11b $${Y}_{2}=\frac{{y}_{2}}{{F}_{0}}=\zeta \left(1-\frac{W}{\beta }\right).$$

The phase center location for beam ports $({x}_{1}.{y}_{1})$ can be also expressed as:

12a $${X}_{1}=\frac{{x}_{1}}{{F}_{0}}={\rho }_{0\left[1-{\text{cos}}({\alpha }{^\prime}+\psi )\right]},$$

12b $${Y}_{1}=\frac{{y}_{1}}{{F}_{0}}={\rho }_{0}{\text{sin}}\left({\alpha }{^\prime}+\psi \right),$$

$${\rho }_{0}=1-\frac{1-{\beta }^{2}}{2(1-\beta {\text{cos}}\alpha )},$$

$${\alpha }{^\prime}={\text{sin}}^{-1}\left(\frac{{\text{sin}}\theta }{\gamma }\right),$$

$$\psi ={\text{sin}}^{-1}\left(\frac{1-{\rho }_{0}}{{\rho }_{0}}{\text{sin}}{\alpha }{^\prime}\right).$$

When the location of beam ports and array ports are determined, a horn-type tapering transmission can be used for connecting the transmission lines to the lens body aiming to provide appropriate matching. Also, the wall side of the lens body is connected to the number of matched dummy ports to make a reflection-less parallel-plate contour. There is no specific requirement for the number of dummy ports. Some designers implement multiple dummy ports while others utilize a single dummy port for each side of the lens body. However, some studies indicate that the number of dummy ports does not change the main beam performance and only may affect the side lobe levels (SLLs)^19,20^. Therefore, the main intention of the beam port, array port, and dummy port design is to provide appropriate reflection and transmission coefficients and SLLs.

## Novel wide-angle Rotman lens beamformer design

The proposed beamforming network suitable for 5G mmWave Advanced Antenna System (AAS) should be at least 8 × 8 array supporting mmWave frequency bands allocated to 5G as defined in Section I steering ± 60° and ± 15° in horizontal and elevation planes respectively. Thus, designing an 8-element array, wideband, and wide-angle (± 60) beamformer with dual-polarization capability is a basic demanding requirement for 5G AAS. A novel Rotman lens is designed to meet the basic beamforming requirements for 5G application. The Rotman lens is modeled and optimized with Matlab. The modeled beamformer is simulated and optimized using the Ansys HFSS electromagnetic simulator package. The design procedure consists of the following steps:

- End-fire single antenna element design for dual-polarization capability.
- Lens body design and optimization for wide-angle steering (± 60).
- Beam and array ports and connected transmission line design.
- Dummy port design.
- Non-uniform array port design for SLL reduction.

### Single element design

The single antenna element suitable for 5G AAS is recommended to be dual-polarized with a half-power beam width of at least 65° and in line with 3GPP mmWave frequency bands. In addition, the single element is preferred to be integrated into the beamforming network to avoid using large numbers of connectors and associated losses. Thus, an end-fire type antenna element is selected for easy integration to the beamforming network as well as its capability to use in dual-polarization configuration. As a consequence, a novel Vivaldi antenna is designed to be utilized in the AAS beamformer. The proposed antenna is fabricated on Rogers 4350B (${\varepsilon }_{r}=3.48$) substrate with a thickness of 0.254 mm. The design methodology, fabrication, and results are comprehensively discussed in Ref.^21^.

**Figure 3:**
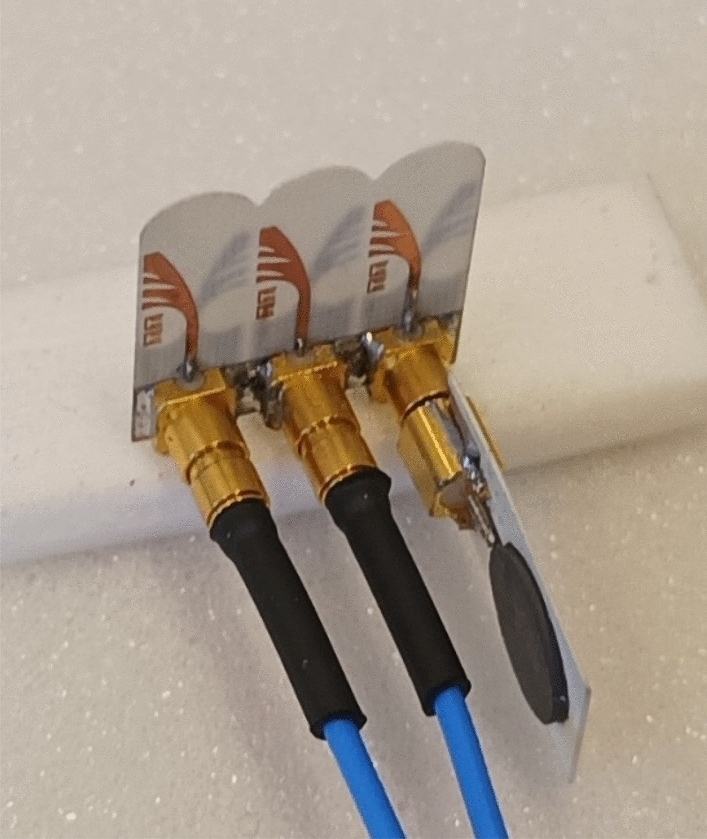
Fabricated Vivaldi antenna array.

The structure of the proposed Vivaldi antenna element is presented in Fig. 3. The antenna has a compact size of 12 × 5.5 × 0.254 mm^3^. Therefore, the space element is d = 5.5 mm if the antenna is used in the array. The proposed antenna can operate in 23–45 GHz covering 5G n257, n258, n259, n260, and n261 frequency bands and exhibit a nearly constant end-fire radiation pattern with a measured gain of more than 5dBi across the whole bandwidth^21^.

### Lens body design

It is intended to design a basic 8-element Rotman lens beamformer between 24 and 30 GHz covering 5G *n257*, *n258*, and *n261* frequency bands for AAS applications. The Rotman lens is designed for fabrication on a dielectric substrate. The dielectric substrate used is Rogers 4350B (${\varepsilon }_{r}=3.48$) with a thickness of 0.254 mm. The design methodology can be presented as a step-by-step process based on the approach detailed in “Rotman lens beamformer design” section.

#### Determine the requirements

As indicated, the operational frequency band is between 24 and 30 GHz. The number of array elements is *N* = 8 and the number of beam ports is assumed to be *M* = 8. The steering angle to meet the AAS requirement is ${\theta }_{0}={60}^{^\circ }$ which is considered to be a wide angle. The 3dB beam width of the *N*-element array is:

13 $$M=\left[\frac{{2\theta }_{0}}{{BW}_{array}}\right]\to {BW}_{array}=\frac{120}{8}={15}^{^\circ }.$$

Considering the array beam width $({BW}_{array}={15}^{^\circ })$, the maximum coverage angle ${\theta }_{0}={60}^{^\circ }$ can be moderated as $\theta =60-15/2={52.5}^{^\circ }$. Thus, assuming the steering angle $\theta ={52.5}^{^\circ }$, the 3 dB beam width of the resultant array can cover the AAS coverage requirement ${(\pm 60}^{^\circ })$.

The element spacing equal to the width of a single antenna element is *d* = 5.5 mm. The substrate parameters are ${\varepsilon }_{r}=3.48$ and *h* = 0.254 mm.

1. Calculate the minimum on-axis focal length ${F}_{0}$ using Eq. (1).
  $${F}_{0}\ge 2 \left(8-1\right)5.5 {\text{ sin}}{52.5}^{^\circ }=61.08\text{ mm.}$$
2. Set the ${y}_{3}={y}_{3({\text{max}})}$ and ${\zeta }_{\text{max}}=0.5$ and calculate the initial parameters $\gamma .\alpha$ using (5), and (3) respectively.
  $${y}_{3({\text{max}})}=19.25\text{ mm}\stackrel{{\zeta }_{\text{max}}=0.5}{\to } \gamma =1.58\to \alpha ={30}^{^\circ }.$$
  The initial $\beta$ can be selected from Fig. 2 as $\beta =0.88$.
3. Optimize the value of $\alpha .\beta$ using (9) and (10) to obtain optimum phase and amplitude performance. In this research work, the genetic algorithm (GA) is employed for numerical optimization using Matlab. The optimized parameters are indicated as:
  $$\alpha ={29.7}^{^\circ } , \beta =0.91.$$
4. Specify the difference in transmission line length for the side array port compared to the center array port (*W*) using (7) as:
  $$W=1.45\text{ mm}$$
  Divide the dimensions by a factor of $\sqrt{{\varepsilon }_{r}}$. The parameters ${F}_{0}. {F}_{1}. W$ are divided to $\sqrt{3.66}$ as the designed dielectric constant is recommended 3.66 for Rogers 4350B^22^.
5. The modeled body lens parameters are summarized in Table 2.

**Table 2:** Designed Rotman lens parameters.

Parameter	F0 (mm)	F1 (mm)	α (deg)	β	ζ	W (mm)
Value	31.93	29.05	29.7°	0.91	0.7	0.76

### Beam and array ports and transmission line design

The phase center location of beam ports $({x}_{1}\cdot {y}_{1})$ can be calculated using (12a) and (12b) as:

$${x}_{1}=25.23\text{ mm }\quad  {y}_{1}=14.4\text{ mm.}$$

Also, the phase center location of array ports $({x}_{2}.{y}_{2})$ can be calculated using (11a) and (11b) as:

$${x}_{2}=0.68\text{ mm } \quad {y}_{2}=14.9\text{ mm,}$$

where the origin is the center of the array port arc. Considering the center location of the ports, the width of the lens port aperture is roughly ${W}_{p}=4.65\text{ mm}$.

The microstrip width (${w}_{1}$) of the feed line can be calculated as^23^:

14 $${w}_{1}=\frac{7.48\times h}{{e}^{\left({Z}_{0}\frac{\sqrt{{\varepsilon }_{r}+1.41}}{87}\right)}}-1.25\times t\approx 0.55\text{ mm,}$$

where ${Z}_{0}$ is the characteristic impedance, *h,* and *t* are the substrate and track thicknesses respectively. Considering the 50 Ω characteristic impedance and proposed substrate parameters, ${w}_{1}\approx 0.55\text{ mm}$. However, the microstrip line width is slightly optimized as ${w}_{1}=0.53\text{ mm}$ in the simulation process.

After determining the phase center location, lens port width, and feed line width, a horn with the appropriate length ${(L}_{p})$ is tapered toward the feed to overcome the impedance discontinuity problem due to connecting the large lens port width to the small feed line width.

According to Ref.^24^, the length of the triangular transition ${L}_{p}$ is suggested to be as follows:

15 $${L}_{p}\approx 4.57{W}_{p},$$

where ${W}_{p}$ is the width of the lens port aperture.

**Figure 4:**
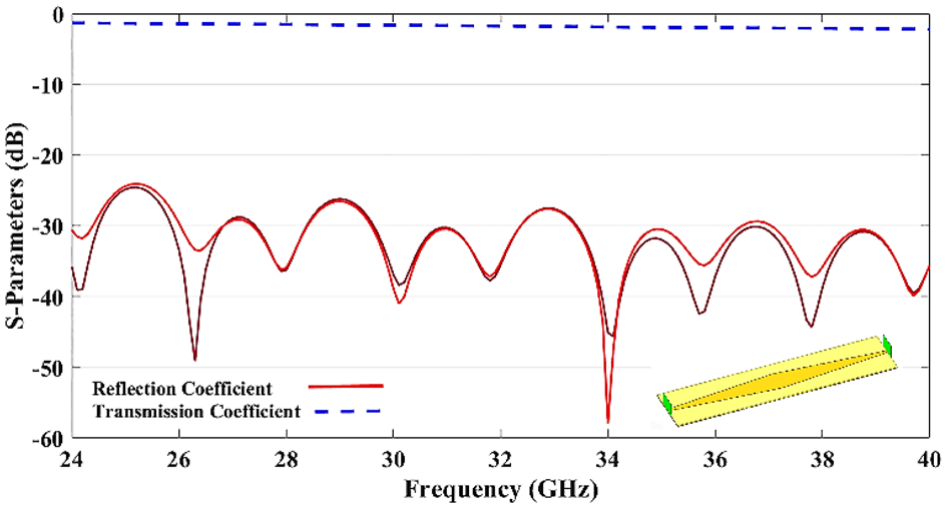
Tapering transition modeling and simulation.

**Figure 5:**
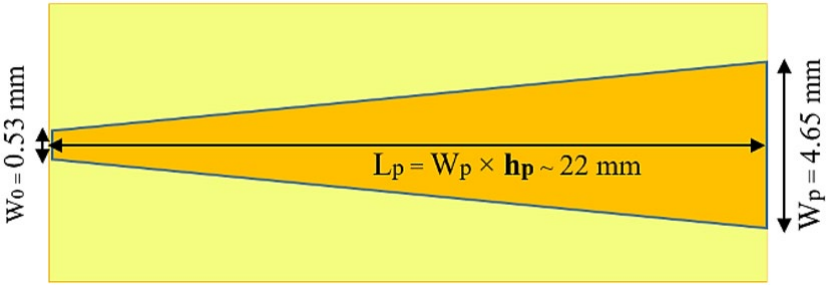
Schematic of the tapering transition.

In this work, the tapering length is optimized based on a model and extracted results as shown in Fig. 4 to obtain optimum matching and insertion loss. As a result, the optimized horn length is ${L}_{p}=22\text{ mm}$ where the number 4.57 in Ref.^24^ is adjusted as ${h}_{p}=4.73$. The schematic of the tapering transition is depicted in Fig. 5.

Due to the very small distance between the ports and easy interconnection purpose, SMPM connectors are used. The impedance matching between the SMPM connector and the microstrip line is very sensitive for mmWave bands. The SMPM connectors are used for the beam ports using the transition procedure as detailed in Ref.^25^.

### Dummy port design

In this design, we employ only a single dummy port on each side of the parallel plate contour with a wide aperture width in order to convene multiple dummy ports and simplify the structure.

The dummy ports are matched using a novel absorber sheet based termination load as described extensively in Ref.^26^.

**Figure 6:**
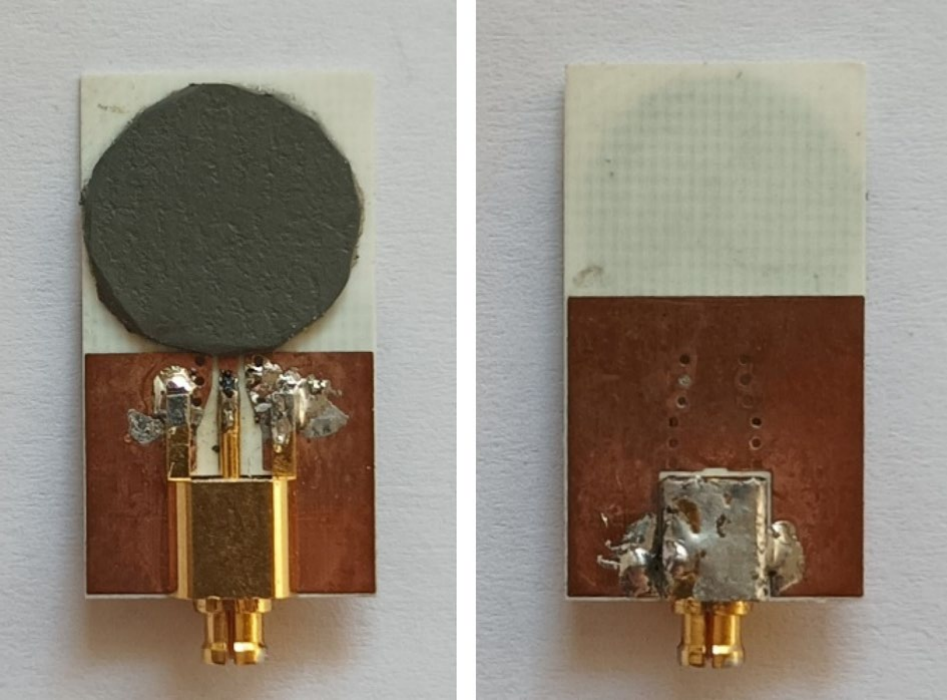
Fabricated SMPM terminator.

The proposed high-performance and cost-effective microstrip termination load is based on the combination of a printed monopole antenna and an absorber sheet as shown in Fig. 6 that can be easily integrated with a microstrip line or used with a connector as a termination load for test measurement. The results show a good impedance matching between 20 and 67 GHz that can be effectively used as loaded dummy ports for mmWave applications.

In this design, this termination load is integrated into the dummy ports to provide good termination and reflection-less side walls of the Rotman lens.

**Figure 7:**
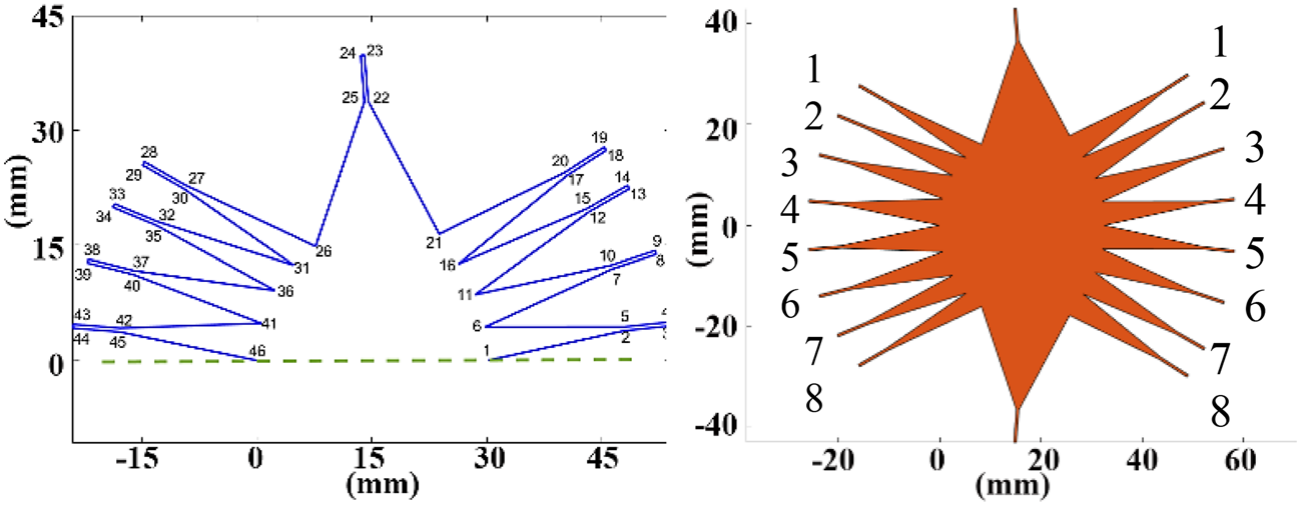
Rotman lens modeling and geometry.

Once the whole Rotman lens parameters are specified, a mathematic-based geometry of the Rotman lens can be generated by Matlab as shown in Fig. 7.

The generated geometry can be imported to full wave simulation packages. Thus, the modeled Rotman lens geometry is imported to Ansys HFSS for simulation and further optimization.

### Non-uniform array port design

The side lobe level (SLL) is a challenging parameter in the Rotman lens due to the unwanted reflections from side walls, beam and array ports, and also dummy ports^19^.

Chebyshev is a famous tapered distribution that can be used to set SLL to a specified value (*s*). In an *N-*element array, the peak value of the Chebyshev polynomial of the order *N − 1* can be expressed as^27^:

16 $${T}_{N-1}\left({z}_{0}\right)={10}^{s/20},$$

where *s* is SLL in dB and ${z}_{0}$ is the main lobe position that can be calculated as:

17 $${z}_{0}={\text{cos}}h\left[\frac{{{\text{cos}}h}^{-1}({10}^{s/10})}{N-1}\right].$$

The half power beam width (HPBW) of the scanning array can be obtained using:

18 $$HPBW={\text{cos}}^{-1}\left[{\text{cos}}\theta -0.443\frac{\lambda }{L+d}\right],$$

$$-{\text{cos}}^{-1}\left[{\text{cos}}\theta +0.443\frac{\lambda }{L+d}\right],$$

where *L* is the array length, *d* is the inter-element space, and $\theta$ is the scanning angle.

In a non-uniform array design, the SLL can be controlled by the amplitude distribution among the elements and there is a tradeoff between SLL and HPBW where by decreasing the SLL, the HPBW is decreased^27–29^.

**Figure 8:**
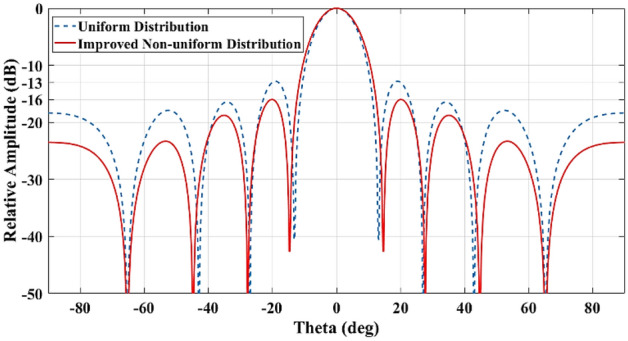
Comparison of SLL for uniform and non-uniform array ports.

In this research work, firstly an improved distribution scheme for the target array is achieved based on a Matlab code aiming to enhance the SLL while the HPBW is almost constant. The resultant $SLL\approx 16\text{ dB}$ as depicted in Fig. 8 and the distribution coefficient is presented in Table 3.

**Table 3:** Improved Chebyshev distribution by Matlab to enhance the SLL while the HPBW is almost constant.

Array port	1 and 8	2 and 7	3 and 6	4 and 5
Amplitude weighting	0.65	0.85	0.89	1
Width of port (mm)	2.9	3.85	4.1	4.65

The improved non-uniform amplitude distribution is applied to the array port by altering the port width. Assuming the relation of power and impedance^22^:

19 $$P=\frac{{V}^{2}}{2Z}.$$

The impedance of microstrip as a function of microstrip width to substrate height $W/h$ is^26^:

20 $$Z=\frac{120\pi }{\sqrt{{\varepsilon }_{eff}}\times \left[\frac{W}{h}+1.393+\frac{2}{3}{\text{ln}}\left(\frac{W}{h}+1.444\right)\right]} \frac{W}{h}>1.$$

The following relation can be extracted for the width of the microstrip line:

21 $$\frac{{P}_{1}}{{P}_{2}}\approx \frac{{Z}_{2}}{{Z}_{1}}\approx \frac{\frac{{W}_{1}}{h}+1.393+\frac{2}{3}{\text{ln}}\left(\frac{{W}_{1}}{h}+1.444\right)}{\frac{{W}_{2}}{h}+1.393+\frac{2}{3}{\text{ln}}\left(\frac{{W}_{2}}{h}+1.444\right)}\approx \frac{{\text{ln}}\left(1+4\left(\frac{h}{{W}_{2}}\right)\right)}{{\text{ln}}\left(1+4\left(\frac{h}{{W}_{1}}\right)\right)}.$$

The width of the array port based on the amplitude coefficient using (21), when the center port width is 4.65 mm can be calculated as indicated in Table 3.

The new Rotman geometry including the non-uniform array ports as indicated in Table 3 is generated for further optimization by the full wave simulation. To this effect, the Rotman lens parameters, and amplitude weighting together with the position of the array ports are optimized using genetic algorithm (GA) by HFSS in terms of phase and amplitude errors and SLLs in the widest angle beam (${52.5}^{^\circ }$) as the worst-case for 24 GHz and 30 GHz as the start and stop operating frequencies. The optimized array ports width and corresponding distribution coefficient are shown in Table 4. The symmetrically oriented array port numbers can be found in Fig. 7.

**Table 4:** Optimized distribution by full-wave simulation.

Array port	1 and 8	2 and 7	3 and 6	4 and 5
Amplitude weighting	0.66	0.87	0.92	1
Array port width (mm)	${W}_{a1.8}=$ 2.88	${W}_{a2.7}=$ 3.94	${W}_{a3.6}=$ 4.17	${W}_{a4.5}=$ 4.61

**Figure 9:**
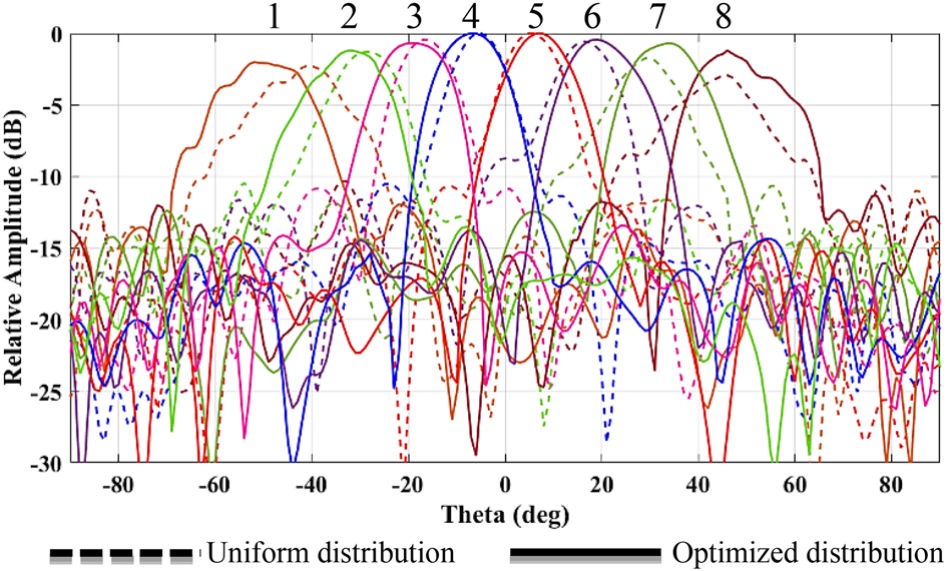
Comparison of SLL for uniform and improved non-uniform array ports.

The simulated resultant radiation patterns by exciting different beam ports for uniform and improved non-uniform distribution of array ports are presented in Fig. 9.

It is clear that applying an optimized non-uniform distribution coefficient improves the SLL as well as the amplitude and phase performances. The minimum SLL for uniform distribution is around 9 dB while it is increased to around 12 dB for optimized distribution.

**Figure 10:**
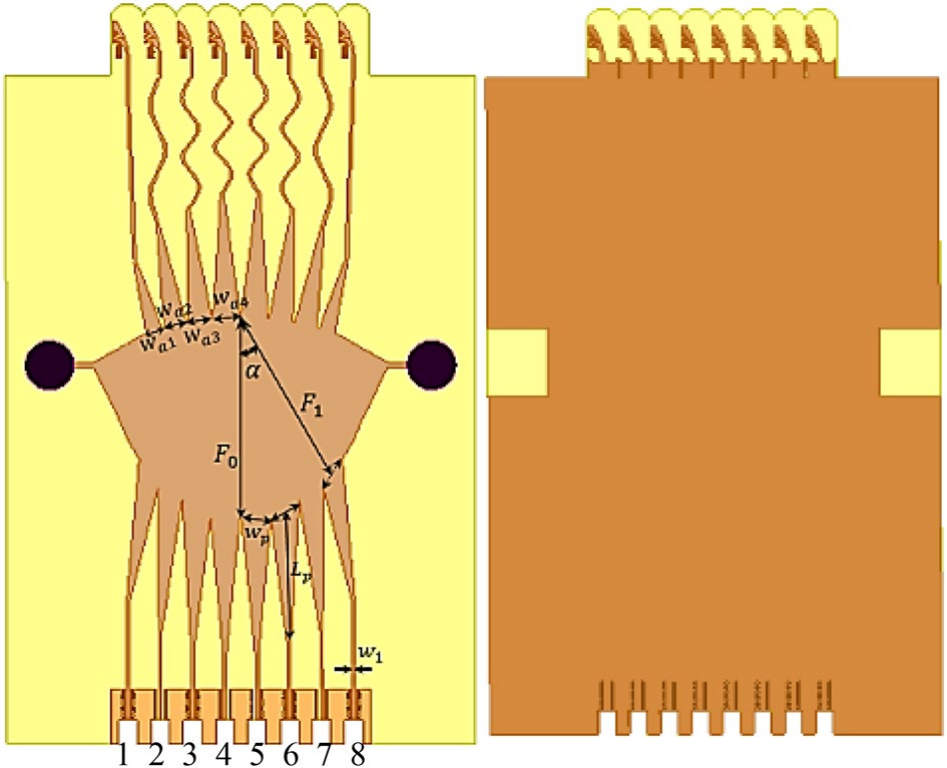
Proposed Rotman lens beamformer structure.

The structure of the final Rotman lens beamforming network with optimized parameters is shown in Fig. 10. Also, Table 5 summarizes the designed and optimized Rotman lens parameters.

**Table 5:** Designed and optimized Rotman lens parameters.

Parameters	Designed value	Final optimized value
${F}_{0}$	31.93 mm	32.66 mm
${F}_{1}$	29.05 mm	29.85 mm
$\alpha$	29.7°	29.81°
$\beta$	0.91	0.913
${w}_{1}$	0.55 mm	0.53 mm
${w}_{p}$	4.65 mm	4.73 mm
${L}_{p}$	22 mm	23.7 mm
${w}_{a1}$	2.9 mm	2.88 mm
${w}_{a2}$	3.85 mm	3.94 mm
${w}_{a3}$	4.1 mm	4.17 mm
${w}_{a4}$	4.65 mm	4.61 mm
W (Difference)	0.76 mm	0.45 mm

It can be concluded that the optimized values are very close to the designed values confirming a good convergence between the optimized simulation parameters and the proposed design procedure.

## Fabrication and measurement results

**Figure 11:**
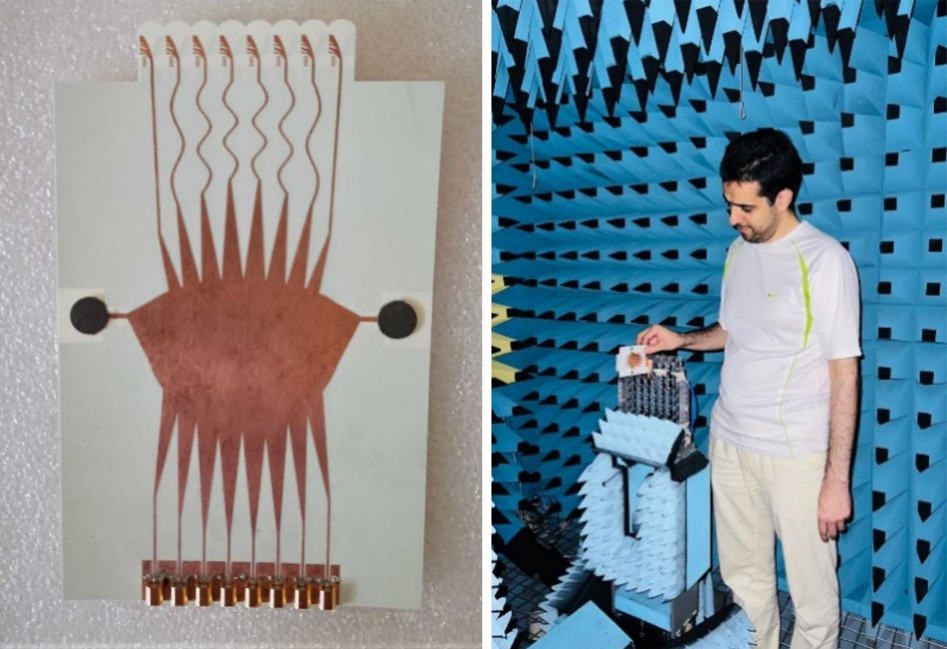
Fabrication and measurement of the Proposed Rotman lens beamformer.

The designed Rotman lens beamformer including 8 beam ports and integrated 8 Vivaldi antennas is fabricated and measured as shown in Fig. 11 to validate the design and simulation results.

The SMPM cables are used for measurement to be compatible with the input connectors. One port is excited at each stage and the other ports are terminated with SMPM terminators as designed in Ref.^26^.

**Figure 12:**
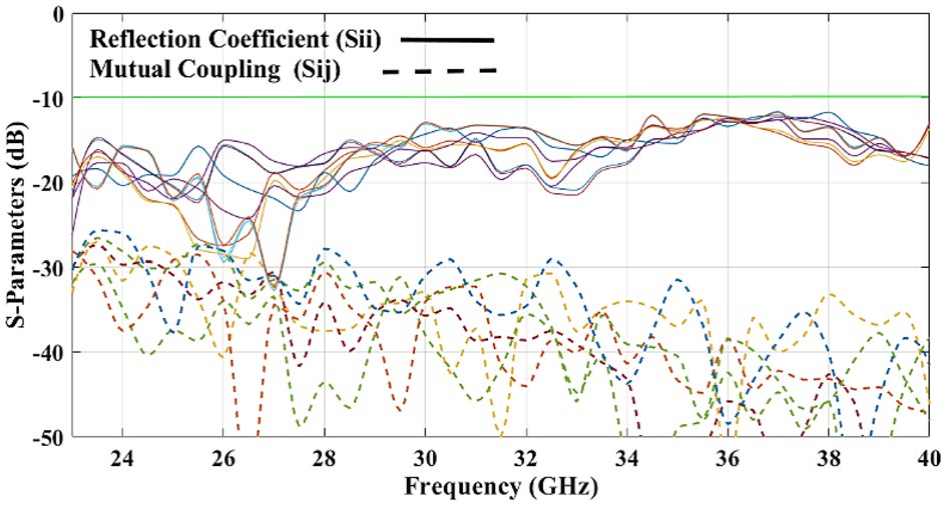
Measured S-parameters of beam ports.

Due to the suitable matching of the tapered beam ports and a large number of S-parameters configurations, the only measured S-parameters are shown in Fig. 12. Reflection coefficient and mutual coupling of the different ports are demonstrated with solid lines and dashed lines respectively.

The measured results show a good matching of the input ports (${S}_{ii}<-15\text{ dB}$) and mutual coupling (${S}_{ij}$) of better than 28 dB for the band 24–40 GHz.

**Figure 13:**
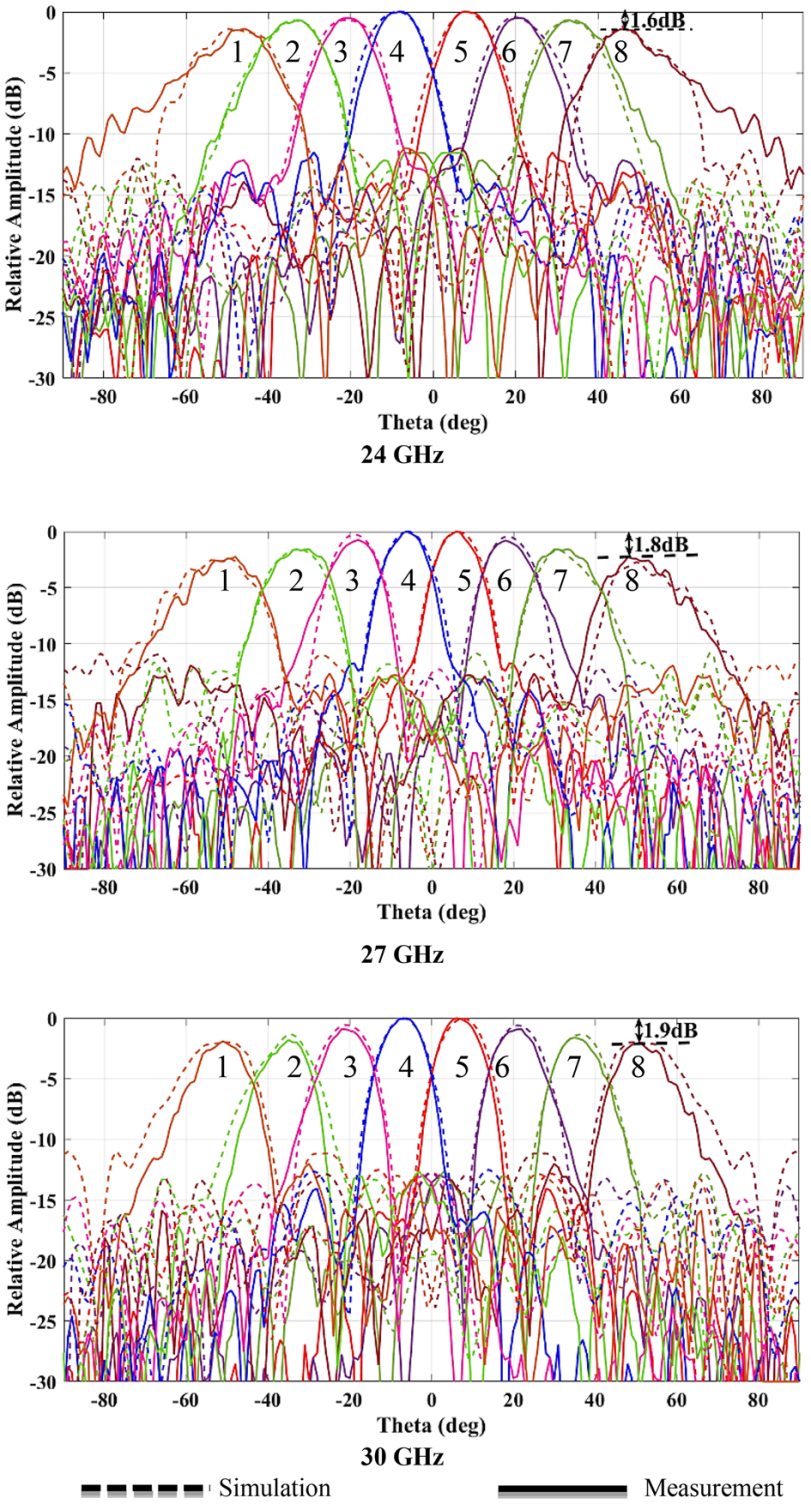
Simulated and measured radiation patterns at 24, 27, and 30 GHz.

**Figure 14:**
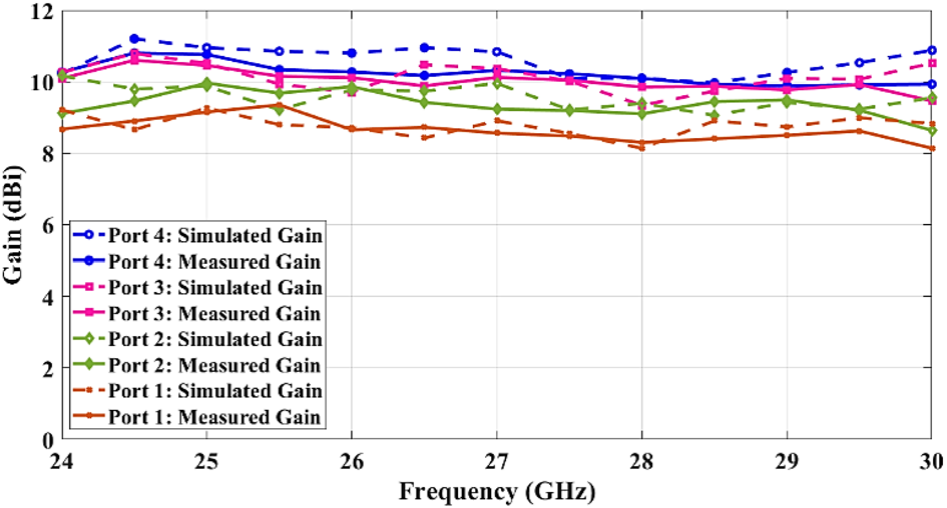
Simulated and measured peak gain versus frequency for different ports.

**Figure 15:**
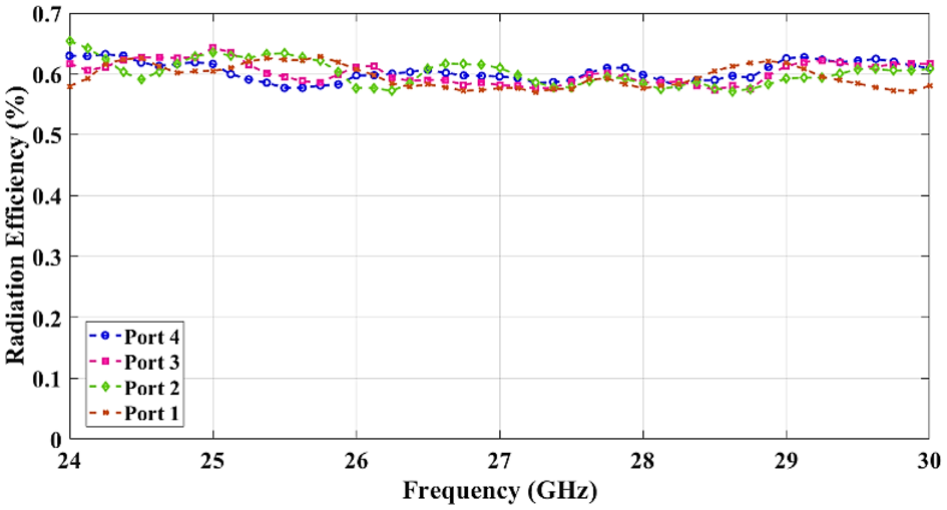
Simulated radiation efficiency versus frequency for different ports.

To test the beamformer performance, the radiation patterns are measured and plotted in comparison with the simulated patterns at 24 GHz, 27 GHz, and 30 GHz in Fig. 13. The simulation and measurement results are depicted in the form of solid lines and dashed lines respectively. The simulated and measured peak gain and simulated radiation efficiency of the Rotman lens beamforming network are also presented for beam ports 1, 2, 3, and 4 in Figs. 14 and 15 due to achieving identical results for the symmetric ports.

The radiation pattern results show a good agreement between simulations and measurements. The beam steering from − 53° to 53° in 15° increments with a slight beam-pointing error of less than 2° is obtained. The radiation patterns show a scan loss at a maximum scanning angle of less than 1.6 dB, 1.8 dB, and 1.9 dB at 24 GHz, 27 GHz, and 30 GHz respectively due to Rotman amplitude error and single element radiation pattern. Also, the scanning directions remain unchanged across the bandwidth as the Rotman lens is a true-time-delay beamformer. The radiation patterns exhibit some ripples for the wide angle which is due to the imperfection and multipath reflections of the anechoic chamber. The proposed Rotman lens offers the SLL better than 10 dB for whole ports that meet the minimum AAS requirements.

The measured peak gain shows the average 10 dBi gain for the center ports (Port 4 and Port 5) and the scan loss of less than 1.9 dB for the wide-angle ports (Port 1 and Port 8). It can be observed that the average radiation efficiency is 62% under every input feed port of the Rotman lens across the bandwidth.

As a result, the proposed Rotman lens provides proper beamforming capability in the wide range of ± 53° to cover ± 60° with the 3dB beamwidth in wide target frequency bands.

The proposed Rotman lens beamformer is compared with some recently recognized Rotman lenses for 5G mmWave applications as summarized in Table 6.

**Table 6:** Performance comparison of the proposed Rotman lens.

Ref.	Type	Possible dual-pol	Freq. band (GHz)	Scan angle (°)	SLL (dB)	Scan loss (dB)
^10^	Butler	No	27.8–30.8	± 45	11	1
^11^	Butler	Yes	26–31.4	± 42	8	3
^12^	Rotman	No	28	± 60	8	3
^13^	Rotman	No	25.5–28.5	± 30	8.5	4.5
This work	Rotman	Yes	24–30	± 60	10	1.9

As can be seen, most of the beamformer designs are restricted to the limited bandwidth and scan angle while the SLL is below 10 dB and the scan loss is almost high for the wide-angle beam. The proposed Rotman lens beamformer in this work offers a wide bandwidth (24–30 GHz) and wide scanning range of ± 60° with SLL > 10 dB and scan loss < 1.9 dB which meets the 5G AAS requirements and exhibits better performances compared to most of the other literary works.

## Possible dual-polarized 2-D configuration

The end-fire antenna element integrated with the beamforming network facilitates dual polarization implementation and possible stacking to obtain 2-D beamforming.

**Figure 16:**
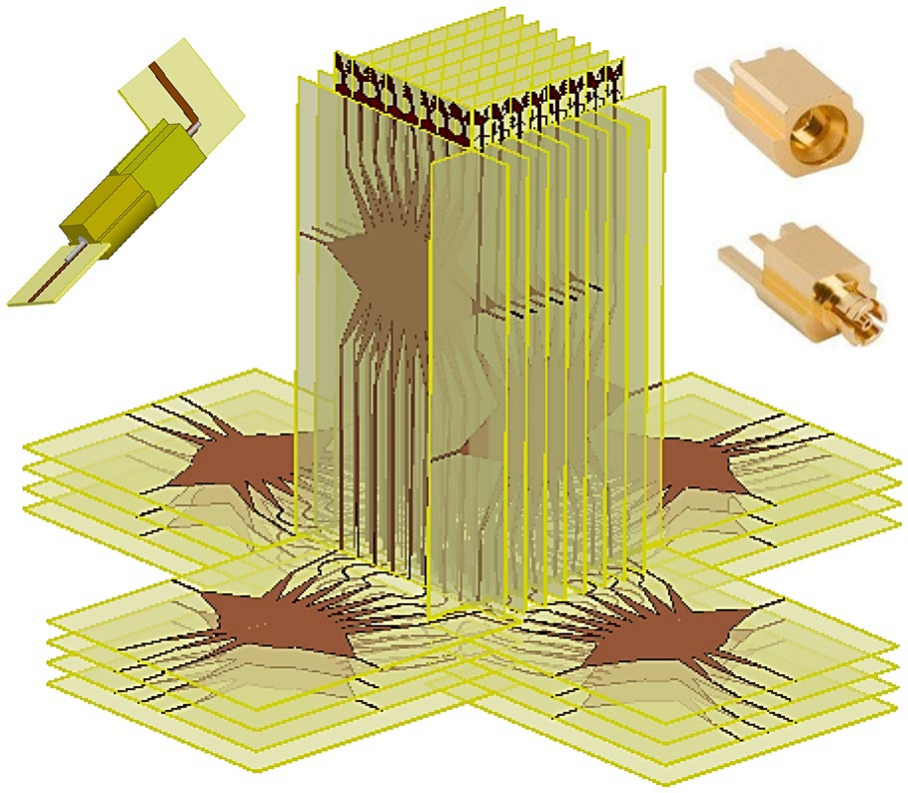
The model of 8 × 8 dual-polarized 2-D AAS.

To design the 8 × 8 dual-polarized 2-D AAS, the proposed Rotman lens beamformers are crossed and vertical to each other as shown in Fig. 16.

To realize the crossing, the extended length of the transmission lines is equal to the self-length of the Rotman lens. 8 proposed Rotman lens beamformers are staked along the *x*-direction and *y*-direction with stacking spacing *d* (Fig. 16).

The first stage of the beamformer can be connected to the second stage of the beamformers via the SMPM connectors to construct the 2-D beamforming network. The recommended steering angle of the second stage of the beamformer is ± 15° (Table 1) which can be generated using a 3-beam port Rotman lens with a step angle of 10°.

## Conclusion

Beamforming is an important part of mmWave technologies to improve the link budget and spectral efficiency and abilities of MIMO and SDMA. The beamforming requirements for 5G mmWave applications indicate the wide-angular coverage of ± 60° and SLL > 10 dB. A wide-angle Rotman lens with a detailed improved design methodology to satisfy the 5G mmWave beamforming is presented. The enhanced SLL is obtained by imposing an optimized distribution on the aperture of the antenna ports. The proposed seamless beamformer and antenna array is fabricated using low-cost PCB technology on Rogers 4350B (${\varepsilon }_{r}=3.48$) substrate with a thickness of 0.254 mm and successfully tested to verify the feasibility of the design methodology. The overall results demonstrate that the proposed beamformer exhibits wideband impedance and radiation characteristics over a bandwidth of 24 –30 GHz and beam-scanning capability over a scan range of ± 60° with SLL > 10 dB and scan loss < 1.9 dB. Dual-polarization and 2-D beamforming configurations of the beamformer are also proposed.

The resulting beamformer features unique characteristics such as wide angular coverage with acceptable SLL and low scan loss over a wide frequency bandwidth of 24–30 GHz covering three standard 5G *n257*, *n258*, and *n261* bands.

The proposed beamforming network is qualified for various mmWave and 5G applications such as AAS, massive MIMO systems, hybrid beamforming systems, remote sensing, and automotive radars.

## Data Availability

All the data required to evaluate the findings of this work is available in the manuscript. Any other additional data related to this work may be requested from the corresponding author.

## References

[1] Azari A, Aliakbarian H (2022). Requirements for 5G applications: A strategic approach. Microw. J..

[2] Asplund H, Astely D, Butovitsch P, Chapman T, Frenne M, Ghasemzadeh F, Hagström M, Hogan B, Jongren G, Karlsson J, Kronestedt F, Larsson E (2020). Advanced Antenna Systems for 5G Network Deployments-Bridging the Gap Between Theory and Practice.

[3] *The 3rd Generation Partnership Project (3GPP)*. www.3gpp.org.

[4] Azari, A., Skrivervik, A. K. & Aliakbarian, H. Design methodology for wideband bowtie patch antenna for 5G mmWave applications. In *2023 17th European Conference on Antennas and Propagation (EuCAP)* 1–4 (2023).

[5] Ahmed I (2018). A survey on hybrid beamforming techniques in 5G: Architecture and system model perspectives. IEEE Commun. Surv. Tutor..

[6] Zhang J, Yu X, Letaief KB (2020). Hybrid beamforming for 5G and beyond millimeter-wave systems: A holistic view. IEEE Open J. Commun. Soc..

[7] Mailloux RJ (2005). Phased Array Antenna Handbook.

[8] Lialios DI (2020). Design of true time delay millimeterwave beamformers for 5G multibeam phased arrays. Electronics.

[9] Dong, J. & Zaghloul, A. I. Implementation of microwave lens for 360-degree scanning. In *2009 IEEE Antennas and Propagation Society International Symposium* 1–4 (2009).

[10] Qin C, Chen F-C, Xiang K-R (2021). A 5 × 8 butler matrix based on substrate integrated waveguide technology for millimeter-wave multibeam application. IEEE Antennas Wirel. Propag. Lett..

[11] Klionovski K (2019). A dual-polarization-switched beam patch antenna array for millimeter-wave applications. IEEE Trans. Antennas Propag..

[12] Eid A, Hester JGD, Tentzeris MM (2020). Rotman lens-based wide angular coverage and high-gain semipassive architecture for ultralong range mm-Wave RFIDs. IEEE Antennas Wirel. Propag. Lett..

[13] Heino M, Icheln C, Haarla J, Haneda K (2020). PCB-based design of a beamsteerable array with high-gain antennas and a Rotman lens at 28 GHz. IEEE Antennas Wirel. Propag. Lett..

[14] Rotman W, Turner R (1963). Wide-angle microwave lens for line source applications. IEEE Trans. Antennas Propag..

[15] Hansen RC (1991). Design trades for Rotman lenses. IEEE Trans. Antennas Propag..

[16] Smith MS (1982). Design considerations for Ruze and Rotman lenses. Radio Electron. Eng..

[17] Mujammami EH, Afifi I, Sebak AB (2019). Optimum wideband high gain analog beamforming network for 5G applications. IEEE Access.

[18] Simon, P. Analysis and synthesis of Rotman lenses. In *Proc. 22nd AIAA Int. Commun. Satell. Syst. Conf. Exhib. (ICSSC)* 3196 (2004).

[19] Darvazehban A, Manoochehri O, Salari MA, Dehkhoda P, Tavakoli A (2017). Ultra-wideband scanning antenna array with rotman lens. IEEE Trans. Microw. Theory Tech..

[20] Penney CW (2008). Rotman lens design and simulation in software (application notes). IEEE Microw. Mag..

[21] Azari A, Skrivervik A, Aliakbarian H, Sadeghzadeh RA (2023). A super wideband dual-polarized vivaldi antenna for 5G mmWave applications. IEEE Access.

[22] *Rogers RO4350B Datasheet*. https://www.rogerscorp.com/-/media/project/rogerscorp/documents/advanced-electronicssolutions/english/data-sheets/ro4000-laminates-ro4003c-and-ro4350b---data-sheet.pdf.

[23] Pozar DM (1998). Microwave Engineering.

[24] Liang Q, Sun B, Zhou G, Zhao J, Zhang G (2019). Design of compact Rotman lens using truncated ports with energy distribution slots. IEEE Access.

[25] Azari, A., Skrivervik, A. K., Aliakbarian, H. & Sadeghzadeh, R. A. High performance low cost transition connectors for 5G mmWave applications. In *International Conference on Millimeter-Wave and Terahertz Technologies* (2022).

[26] Azari A, Skrivervik AK, Aliakbarian H (2023). High performance low cost microstrip termination load for mmWave applications. Electron. Lett..

[27] Balanis CA (2016). Antenna Theory Analysis and Design.

[28] Toan TT, Tran NM, Giang TVB (2017). A feeding network with Chebyshev distribution for designing low sidelobe level antenna arrays. VNU J. Sci..

[29] Nissanov U (2021). THz equal and unequal 1 to 8 T-Junction power dividers. Sens. Int..

